# How can policies to reduce obesity be more effective and equitable following the COVID-19 pandemic?

**DOI:** 10.1093/eurpub/ckac131.239

**Published:** 2022-10-25

**Authors:** C Gallagher Squires, A Isaacs, P Coleman, C Hawkes

**Affiliations:** Centre for Food Policy, City, University of London, London, UK

## Abstract

Despite being a public health priority in the UK for decades, rates of childhood obesity are continuing to rise along a highly unequal socioeconomic gradient. COVID-19 has radically changed daily life, altering the economy, work, social lives and engagement with the food environment. This research aimed to investigate families’ lived experiences of food in the COVID-19 pandemic to understand how policies to reduce obesity can be more effective and equitable. We conducted a remote longitudinal qualitative study, engaging 62 parents of school or nursery age children in England. Participant demographics were diverse in terms of socioeconomic status and ethnicity. Participants took part in semi-structured interviews and photo-elicitation three times at six-month intervals between October 2020 and December 2021. The role of food in day-to-day life shifted in the context of changes brought about by the pandemic. Whether this led to healthy or unhealthy food practices was shaped by socioeconomic resources. Food became a key source of pleasure in daily life as social and leisure activities were restricted in lockdowns. As schools and childcare closed, when this work fell on one parent food became a source of relentless work and parents sought more convenient options. Those with financial resources were able to access healthier convenient options (e.g. meal boxes), while low incomes restricted parents to low-cost options (e.g. ready meals and fast food). For those experiencing financial insecurity, food became a financial management strategy and parents sought discounts and promotions to save money to cover other non-food essentials. These contexts have the capacity to occur again both on a large scale (e.g. disruptions to the food system) and in the context of an individual’s lifetime (e.g. ill health or job loss). Policy now has a window of opportunity to implement learnings from this period and shape obesity prevention policy to be more effective and equitable.

**Key messages:**

• COVID-19 has revealed the multiple resources and systems of support that underpin families’ ability to eat well and, when disrupted, can limit capacity to procure and prepare nutritious foods.

• Policies designed to improve diets must consider the multiple roles food plays in everyday life, beyond just a source of nutrition, to ensure actions are effective and equitable.

